# Geographic analysis of the variation in the incidence of ADHD in a country with free access to healthcare: a Danish cohort study

**DOI:** 10.1186/s12942-015-0018-4

**Published:** 2015-08-22

**Authors:** Kathrine Bang Madsen, Annette Kjær Ersbøll, Jørn Olsen, Erik Parner, Carsten Obel

**Affiliations:** Department of Public Health, Aarhus University, Bartholins Allé 2, 8000 Aarhus C, Denmark; National Institute of Public Health, University of Southern Denmark, Øster Farimagsgade 5A, 1353 Copenhagen K, Denmark

**Keywords:** Attention deficit hyperactivity disorder, Incidence proportion, GIS, Spatial analysis, Geographic variation, Diagnostic resources

## Abstract

**Background:**

The prevalence of citizens diagnosed with Attention Deficit Hyperactivity Disorder (ADHD) has risen dramatically over the past decades in many countries, however, with large variations. Countries such as Denmark with centrally organized well fare systems, free access to health services and individual tracking based on unique personal identification may in particular contribute to our understanding of the reasons for this increase. Based on Danish registers we aimed to examine the geographical patterns of the distribution of ADHD diagnosis and medication use and explore the association with access to diagnostic services, diagnostic culture, neighbourhood socioeconomic status and municipal spending on health care for children.

**Methods:**

We combined information on registered diagnosis of ICD-10 Hyperkinetic Disorder and ADHD medication use in a Danish register-based cohort of children born between 1990 and 2000. We mapped incidence proportions of diagnoses and medication use within the 98 Danish Municipalities. Global and local clustering of ADHD was identified using spatial analysis. Information on contextual factors in the municipalities was obtained from national registers. The associations between the incidence of ADHD and contextual factors were analysed using Bayesian spatial regression models.

**Results:**

We found a considerable variation in the incidence of ADHD across the municipalities. Significant clustering of both high and low incidence of ADHD was identified and mapped using the local Moran’s I. Clustering of low incidence of diagnosis and medication use was observed in less populated areas with limited diagnostic resources and in contrast clustering of high incidence in densely populated areas and greater diagnostic resources. When considering the spatial autocorrelation between neighbouring municipalities, no significant associations were found between ADHD and access to diagnostic services, different diagnostic culture, socioeconomic status at municipality level or the municipal spending on health care for children.

**Conclusions:**

A large geographical variation of ADHD in the municipalities was observed despite tax-financed and free access to healthcare. Although not statistically significant, results indicate that accessibility to diagnostic resources might explain some of the variation in ADHD incidence. In contrast to US studies the observed variation was not statistically associated to contextual factors in terms of SES, municipal spending on health care for children or differences in diagnostic practices.

## Background

Attention Deficit Hyperactivity Disorder (ADHD) is a behavioural disorder characterised by inattentiveness, hyperactivity and impulsiveness. In most countries it is the most commonly diagnosed childhood behavioural disorder with an estimated prevalence of 3–5 % among 6 to 12-year old children [1, 2]. The causal pathways of ADHD are complex as both inherited and non-inherited factors contribute and their effects are interdependent [3]. ADHD is a clinically heterogeneous disorder that is associated with a considerable economic burden to society, stress to the affected families, and adverse academic and vocational outcomes to some of the affected children [4]. The recorded prevalence of ADHD has been increasing for the last decades in a number of countries including Denmark; the same tendency has been observed for many other paediatric psychiatric conditions such as Autism Spectrum Disorders (ASD), Obsessive–Compulsive Disorder and Tourette’s syndrome [5, 6]. However, the increase in prevalence of ADHD is not homogenous within countries or regions. In Norway, the diagnostic prevalence of ADHD by county among children aged 6–12 years ranged from 1.1 to 3.4 %, similar differences are found in Sweden and large variations between regions and states are found in the USA [7–12]. Some studies report that residing in rural and semi-rural areas is associated with reduced prevalence of ADHD diagnosis and health service use [13–15].

It has been debated whether this increase in diagnosis and treatment of childhood psychiatric disorders reflects a true increase in the incidence of mental health problems in children, changes in their impact or simply an increase in recognition and help-seeking behaviour. This increase in diagnosis and treatment of psychiatric disorders may be related to contextual changes in society and an improved understanding of the relative contribution of these factors has become of major public interest. A recent study found a reduction over time in parent and teacher perceived levels of behavioural problems in pre-adolescent children in Great Britain concurrent with the increase in diagnosis and treatment; this suggests that the increased prevalence may be due to contextual factors rather than an increase in behavioural problems [16].

Contextual factors causing an increase in diagnosis and treatment could be related to changes in access to diagnostic facilities/number of specialists, diagnostic culture, community resources such as socioeconomic status (SES) and spending on primary health care for children; these factors have been suggested to be associated with geographical differences [8, 17–19]. Examining these contextual factors in relation to the geographical distribution of ADHD may provide a clue to the causal pathway of the increasing prevalence of ADHD. Denmark and other Nordic countries are unique for examining this because it is possible to do a complete follow-up on diagnosis and residence of all citizens as they are registered with a personal identification number.

Studies of factors related to access to diagnostic facilities have found that having a diagnosis of ADHD or taking medication for ADHD was associated with the number, age, and specialisation profile of physicians within states and counties in the US [8, 17]. In Norway, the considerable geographical variation in prevalence of ADHD could be explained by the very decentralised Norwegian specialist health services for children where many institutions treat a very small number of children in each diagnostic group [7].

Psychiatric diagnoses are based on descriptive criteria, interviews and observations; any diagnosis in psychiatry includes an interpretation and decision-making by a professional [20]. A recent Danish study found that the behaviour of specialist physicians varied considerably across hospitals and that the prescribing behaviour affected the probability that a child would receive ADHD medication [21]. Danish hospitals use the WHOs International Classification of Diseases (ICD-10) while the concept of ADHD is from the American diagnosis system DSM-IV (now DSM-5) [22, 23]. The inclusion criteria are far more narrow within the former system, reflected in comparison studies reporting an ICD-10 Hyperkinetic Disorder (HKD) prevalence of 1–3 % and a DSM-IV ADHD prevalence of 4–8 % [24]. Changes in diagnostic practices with a more pronounced clinical use of the DSM criteria in countries like the UK and Germany may very well account for part of the observed increase [25]. The relative use of the two diagnostic systems may also explain some of the variation observed within countries.

An association between family socioeconomic disadvantage and childhood ADHD has been established at the individual level [12, 18, 19], and one study found that the geographical variation in treatment prevalence to some extent was attributable to measured socioeconomic differences at the population level [17]. Hence, SES of a geographical area might be important when considering the risk of being diagnosed with ADHD. The impact of SES for a diagnosis of ADHD is consistent with findings on a wide range of health outcomes. US studies have found that children residing in fortunate SES areas had an increased risk of being diagnosed with autism [26, 27]. The mechanisms underlying the associations between SES and health outcomes are unknown, but SES is probably a good proxy for local resources and the availability of health-related information [27].

Most studies addressing the rise and geographical variation in prevalence of ADHD have been conducted in the US; few have addressed European contexts. The associations between ADHD and access to diagnostic facilities, diagnostic culture and community SES may be very different in a country with free access to qualified health care and economic equality.

In the present study we performed exploratory spatial analysis to examine if the following contextual factors were associated with the risk of ADHD diagnosis or treatment: access to paediatric psychiatrists and psychiatric hospitals, average SES in the community, spending on primary health care for children and diagnostic culture in the public psychiatric hospitals. We used administrative data from Denmark to identify spatial patterns of ADHD that may drive the increase in recorded incidence.

## Results

### Incidence of ADHD

The total number of children with ADHD was 8218 of which 6798 children had a hospital diagnosis, 6693 children redeemed medication, and 1420 children redeemed medication but did not have a registered diagnosis (treated by private practicing paediatric psychiatrists). In all of 750,512 children were born in the period from 1990 to 2000 in Denmark (Table 1).

**Table 1:** ADHD cases in the study population

Hospital diagnosis	Medication	
	No	Yes
No	742,294	1420
Yes	1525	5273

The incidence has been steadily increasing with each birth year. The incidence proportion increased from 0.36 % (95 % CI 0.31; 0.41) in the 1990 birth cohort to 2.58 % (95 % CI 2.46; 2.70) in the 2000 birth cohort (Table 2).

**Table 2:** Incidence proportions in the study population by birth year

Birth cohort	ADHD cases	Births	Incidence proportion (%)	95 % CI
1990	242	66,623	0.36	0.31; 0.41
1991	327	66,791	0.49	0.44; 0.55
1992	371	69,669	0.53	0.48; 0.59
1993	451	68,898	0.66	0.59; 0.72
1994	557	70,999	0.79	0.72; 0.85
1995	662	70,522	0.94	0.87; 1.01
1996	730	67,595	1.08	1.00; 1.61
1997	819	67,134	1.22	1.14; 1.31
1998	1028	65,092	1.58	1.49; 1.68
1999	1366	64,341	2.12	2.01; 2.24
2000	1665	64,630	2.58	2.46; 2.70

**Fig. 1:**
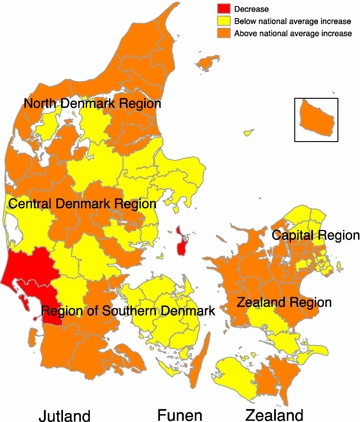
Relative increase in ADHD diagnosis and medication use above or below national average level

The average national incidence proportions were computed for the children born in the period 1998–2000 and in the period 1990–1992. The average national incidence proportion in the 1998-2000 birth cohort was 4.4 times higher (95 % CI 4.1; 4.8) than the incidence proportion in the 1990–1992 birth cohort resulting in a significant national increase in incidence. Figure 1 displays the geographical areas in which the incidence proportion has increased below or above national average increase when comparing the 1990–1992 and 1998–2000 birth cohorts. Three municipalities have experienced a decrease in incidence proportion when comparing the two birth cohorts.

All indicators of ADHD vary considerably across municipalities. The mean incidence proportion of overall ADHD was 1.19 %, ranging from 0 to 2.87 % in the municipalities (Table 3).

**Table 3:** Summary statistics of ADHD incidence proportion and contextual factors

	Mean	SD	Min	Max
Incidence proportion of hospital diagnosis (%)	0.98	0.49	0	2.74
Incidence proportion of medication use (%)	0.96	0.44	0	2.47
Incidence proportion of medication use and no hospital diagnosis (%)	0.20	0.14	0	0.77
Incidence proportion of overall ADHD (%)	1.19	0.52	0	2.87
Average household income (1000 DKK)	480	80	369	788
Municipal spending on health care for children (1000 DKK/child)	0.77	0.18	0.54	1.34
Incidence proportion of conduct disorder F.91 (%)	0.18	0.14	0	0.86
Incidence proportion of conduct disorder F.92 (%)	0.16	0.13	0	0.59

### GIS mapping

**Fig. 2:**
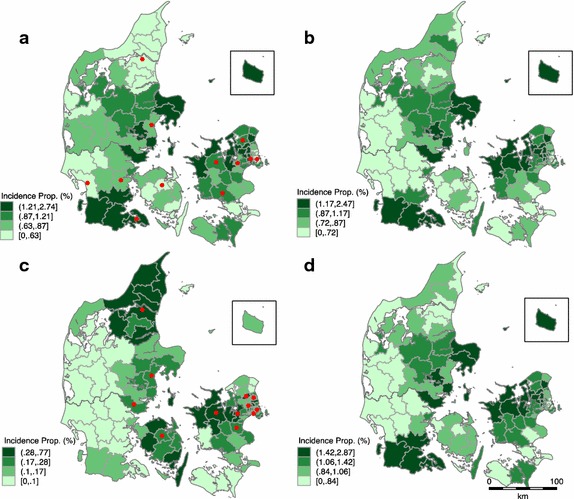
Incidence proportion (%) in children aged 0–11 years old born from 1990 to 2000 of **a** diagnosis, **b** medication use, **c** medication use and no registered diagnosis, **d** overall ADHD by municipality. The incidence proportions are split into quartiles. *Red points* in map **a** and **c** respectively show the public and private diagnostic facilities in the municipalities

Four maps were constructed based on the incidence proportion of ADHD in each municipality using the previously mentioned indicators of ADHD: (a) all children with a diagnosis, (b) all children redeeming medication, (c) children redeeming medication but without a registered diagnosis, (d) all children with a diagnosis and/or redeeming medication (Fig. 2). The public diagnostic facilities in the municipalities are shown as points in map (a), and the private diagnostic facilities are shown in map (c).

Within 98 municipalities in Denmark, 12 paediatric psychiatric wards are placed in 12 different municipalities and 16 private paediatric psychiatrists are practicing in 12 different municipalities. In all, diagnostic facilities are available in 18 municipalities (Fig. 2 map a and c).

### Spatial autocorrelation and cluster identification

Global Moran’s I values indicated that with all four indicators of ADHD the data was not randomly distributed geographically. The analyses suggested significant presence of general tendencies to cluster for all four indicators of ADHD, however, values were very small (Table 4).

**Table 4:** Global Moran’s I statistic of the incidence proportion of the four indicators; diagnosis, medication use, medication use and no diagnosis and overall ADHD

Variables	I	E (I)	Sd (I)	Z	p value
Diagnoses	0.058	−0.010	0.017	3.945	0.000
Medication	0.049	−0.010	0.017	3.426	0.000
Medication no diagnoses	0.090	−0.010	0.017	5.855	0.000
Overall ADHD	0.062	−0.010	0.017	4.172	0.000

**Fig. 3:**
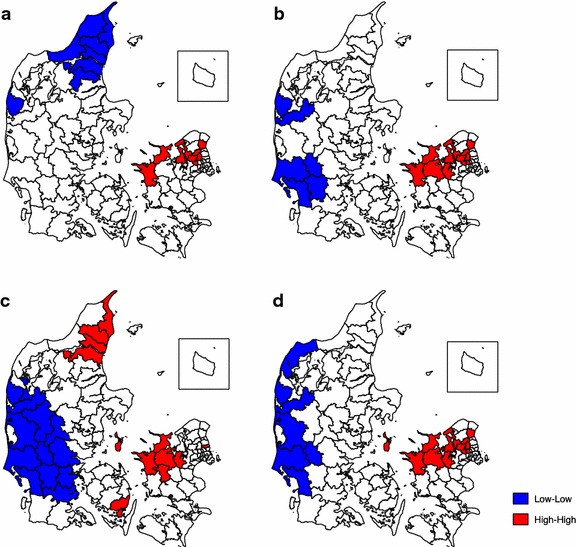
Local Moran’s I clustering of the incidence proportion of **a** diagnosis, **b** medication use, **c** medication use and no registered diagnosis, **d** overall ADHD by municipality (*red* hot spots, *blue* cold spots)

Figure 3 displays the results of the local Moran’s I analyses. In all four analyses both “hot” and “cold” spots were identified. These clustered spots were statistically significant at a 5 % level. Regarding the incidence proportion of diagnosis (map a) a large cluster of high values was found in the northwestern part of the Zealand Region expanding to the western part of the Capital Region. A cluster of low values covered a large part of the North Denmark Region. Looking at the incidence proportion of overall medication use (map b) different clusters of low values emerged. Two “cold” spots were identified, one in the Region of Southern Denmark and one in the Central Denmark Region, both in the western part of Denmark. However, the pattern of clustering of high values was not considerably different from the pattern of the diagnostic incidence proportion. If we only look at the children who use medication but do not have a registered diagnosis (map c) three clusters are significant. Hot spots are found in the North Denmark Region covering almost the entire region and in the northwestern part of the Zealand Region and also in a small part of Funen. One “cold” spot covers almost the entire part of southern Jutland moving upwards covering the western part of Jutland. Looking at all the cases (diagnosis and/or medication) (map d) the clustering of high values only varies a little and the clustering of low values remains in the western part of Jutland. The area with clustering of low incidence has on average a population density of 54 people per km2 (ranging from 20 to 145 people per km^2^ in the municipalities) and the clustering of high incidence is covering an area with an average population density of 175 per km^2^ (ranging from 84 to 403 people per km^2^).

### Non-spatial and spatial regression analysis

The non-spatial logistic regression analysis of overall ADHD, diagnosis, and medication use without diagnosis showed considerable overdispersion with 12.7, 15.0 and 6.4, respectively. The initial non-spatial analysis without adjustment for overdispersion was highly significant for the explanatory variables for each of the three outcomes (Table 5). Adjustment for overdispersion does not affect the parameter estimates (odds ratio (OR)), but results in larger standard errors and wider confidence intervals of the parameter estimates. The analysis adjusted for overdispersion resulted in non-significance of the explanatory variables for the three outcomes except municipal spending for the outcome overall ADHD with *p* value = 0.045 (Table 5).

**Table 5:** Summary statistics of parameters in the non-spatial and spatial regression model for outcomes ADHD, diagnosis and medication use given by odds ratio (OR), 95 % confidence interval (95 % CI) for the non-spatial analysis and 95 % credible interval (95 % CI) for the spatial analysis

Variable^a^	Non-spatial					Spatial	
	No adjustment for overdispersion			Adjustment for overdispersion			
	OR	95 % CI	p value^$^	95 % CI	p value^$^	OR 95 % CI	
ADHD							
Family income							
Low versus high	0.94	0.88; 0.99	<0.001	0.75; 1.17	0.40	0.94	0.76; 1.14
Medium versus high	0.87	0.81; 0.92		0.70; 1.07		0.96	0.81; 1.14
Municipal spending							
Low versus high	1.15	1.08; 1.22	<0.001	0.93; 1.43	0.05	1.03	0.88; 1.22
Medium versus high	1.29	1.22; 1.36		1.06; 1.56		1.05	0.89; 1.23
Conduct disorder							
Low versus high	0.97	0.92; 1.03	0.005	0.80; 1.18	0.66	1.00	0.80; 1.25
Medium versus high	1.07	1.00; 1.14		0.85; 1.34		1.07	0.89; 1.30
Absence of hospital/child psychiatrist	1.14	1.08; 1.20	<0.001	0.95; 1.37	0.16	1.14	0.97; 1.33
	Estimate					Estimate	95 % CI
Dispersion parameter	12.7						
Spatial correlation, ρ						0.69	0.40; 0.90
Spatial variation, τ^2^						0.21	0.15; 0.30

	OR	95 % CI	p value^$^	95 % CI	p value^$^	OR	95 % CI
Medication no hospital diagnosis							
Family income							
Low versus high	1.15	0.98; 1.34	0.07	0.77; 1.70	0.66	0.97	0.70; 1.36
Medium versus high	0.98	0.84; 1.13		0.67; 1.43		1.10	0.83; 1.46
Municipal spending							
Low versus high	1.11	0.95; 1.30	0.02	0.74; 1.64	0.54	1.01	0.77; 1.33
Medium versus high	1.22	1.06; 1.40		0.86; 1.74		0.99	0.77; 1.27
Conduct disorder							
Low versus high	1.00	0.86; 1.15	0.02	0.69; 1.45	0.55	1.01	0.68; 1.48
Medium versus high	1.20	1.02; 1.42		0.79; 1.83		1.20	0.87: 1.69
Absence of a child psychiatrist	0.82	0.71; 0.94	0.006	0.57; 1.17	0.28	0.94	0.70; 1.26
	Estimate					Estimate	95 % CI
Dispersion parameter	6.4						
Spatial correlation, ρ						0.84	0.62; 0.96
Spatial variation, τ^2^						0.41	0.27; 0.63

	OR	95 % CI	p value^$^	95 % CI	p value^$^	OR	95 % CI
Diagnosis							
Family income							
Low versus high	0.92	0.86; 0.99	<0.001	0.70; 1.21	0.49	0.98	0.77; 1.23
Medium versus high	0.86	0.80; 0.92		0.67; 1.10		0.94	0.77; 1.13
Municipal spending							
Low versus high	1.17	1.09; 1.25	<0.001	0.91; 1.51	0.12	1.05	0.87; 1.26
Medium versus high	1.28	1.21; 1.36		1.01; 1.63		1.05	0.87; 1.25
Conduct disorder							
Low versus high	0.95	0.90; 1.01	0.06	0.75; 1.20	0.83	0.95	0.73; 1.22
Medium versus high	1.02	0.95; 1.09		0.78; 1.33		1.02	0.82; 1.25
Absence of a hospital	1.25	1.18; 1.33	<0.001	0.98; 1.59	0.07	1.36	0.80; 1.66
	Estimate					Estimate	95 % CI
Dispersion parameter	15.0						
Spatial correlation, ρ						0.77	0.53; 0.94
Spatial variation, τ^2^						0.27	0.19; 0.39

^$^Overall p-value for the variable

^a^Family income: average yearly total family income. Municipal spending: average municipal spending on primary health care for children. Conduct disorder: percent of children with ICD-10 F91 and F92 diagnoses. All three explanatory variables are categorized into three groups of equal size (33.3 %), low, medium and high

The Bayesian CAR analysis showed no significant effect of any of the explanatory variables for the three outcomes (Table 5).

## Discussion

We found considerable differences in the incidence of documented ADHD in the Danish municipalities ranging from 0 to 2.87 %. This variation is consistent with findings in other western countries [7–9, 12]. The clustering of low incidence proportions is located in less populated areas and in contrast clustering of high incidence proportions is located in densely populated areas. This is not exclusively a Danish phenomenon but is also described in studies from other western countries where diagnosis of ADHD and medication use is less prevalent in rural areas [9, 15].

The recruitment of doctors to less populated areas is known to be difficult and lower incidence of ADHD in rural regions may point to differential healthcare access. Our results indicate that accessibility to diagnostic services is of some importance to the variation in incidence, though not statistically significant (Fig. 2 map a). The locations of privately practicing paediatric psychiatrists are highly correlated with location of psychiatric hospitals; this results in long distances to diagnostic resources in some areas. Since the nineties, there have been 14 paediatric psychiatric wards distributed with at least one in each of the five Danish regions. There are three paediatric and adolescent psychiatric wards in the northern part of the Zealand Region as well as in the Capital Region. The relatively large capacity in the area of paediatric and adolescent psychiatry might explain the clustering of high incidence proportions in these geographical areas. In the western part of Jutland we find clustering of low incidence proportion of both diagnosis and medication use. A few municipalities in this area have even experienced a decrease in incidence of ADHD, which is opposite to the rest of the country. Very few public and no private diagnostic resources are available in the western part of Jutland. Hence, even in a country with free access to healthcare population density and access to diagnostic services might explain the clustering of both high and low incidence of ADHD. In Norway, which is similar to Denmark considering health care and well fare, similar results are found although there are much greater geographical distances [7]. The results are also consistent with findings in US studies despite very different well fare systems [8, 17]. However, considering the incidence of ADHD in all the municipalities, we did not find a statistically significant association to access to diagnostic services when considering the spatial correlation. This is perhaps a result of the relatively low statistical power due to the number of municipalities with diagnostic services (18 out of 98 municipalities). It is possible that access to diagnostic services is of greater importance in the parts where we found clustering than in the rest of the country.

It could also be argued that the large variation in incidence of ADHD between municipalities is a reflection of both under- and over-diagnosing resulting from different diagnostic cultures. A scientific and public debate is ongoing discussing whether ADHD is over-diagnosed in children. Previous studies have suggested that both under-diagnosis and over-diagnosis occur routinely in ADHD [28–30]. Hyperactivity is common, but its diagnosis is still controversial, with two contending approaches: ADHD from DSM IV and hyperkinesis from ICD-10. The concept of ADHD predicts higher rates, but its use may lead to overmedication. Hyperkinesis usefully indicates benefits from medication, but clinics may lead to detection of far fewer cases and thus the possibility of under-diagnosis. It has never been shown whether this lower rate results from hyperkinesis criteria or from the difference in methods used to detect hyperactivity [31]. The validity of the ADHD diagnosis in the Danish Psychiatric Central Register has previously been investigated. However, very few [23] records were examined, of which 89 % were in agreement with a full diagnosis of ADHD according to the DSM-IV; the remaining 11 % lacked one symptom to meet the diagnostic criteria [32]. Although the predictive value of diagnoses in hospitals may be high, it does not clarify the problem of undetected cases in the population.

We explored whether the incidence of ADHD could be affected by different diagnostic cultures by examining the variation in the incidence of the differential diagnosis conduct disorder but we did not find an association; this could be due to an inadequate measure of differences in diagnostic practices. A recent Danish study found considerable variation across hospitals in treatment behaviour of specialist physicians within ADHD [21]. Diagnostic practice is probably far more complex than just distinguishing between the two diagnoses ADHD and conduct disorder. Also, decisions to identify and treat children with ADHD often involve not just the opinion of doctors and patients but also teachers, school psychologists, and parents. Differences in the knowledge and values of other stakeholders may influence prevalence of ADHD [17]. Studies suggest that schoolteachers play an important role in the identification of children with ADHD [33, 34]. It has even been suggested that regional variations in the prescribing of medication for ADHD may be due at least in part to variations in the likelihood of a teacher suggesting the diagnosis of ADHD [33].

Cultural influences related to family’s residence could also affect ADHD prevalence as seen in ASD. The hypothesis is that in geographical areas with high ASD prevalence, the sharing of information on autism between parents increases community awareness about signs and symptoms. This results in children living in very close proximity to a child previously diagnosed with ASD are more likely to be diagnosed with ASD [35]. Parents’ willingness to accept stimulant treatment of their children may also vary geographically, reflecting different beliefs and values about medical treatment or behavioural disorders.

We found no associations with municipality level resources in terms of SES and spending on health care for children. This is in contrast to a study from the US reporting that children living in high-income household areas had a higher incidence of physician-diagnosed ADHD [36]. Another American study on the geographical variation in the diagnosing of autism found that individuals were more likely to be diagnosed with autism when they moved into well-resourced neighbourhoods [27]. An important difference is that the health care system in Denmark is tax financed allowing free and equal access to diagnostic services and thereby the individual household income would be of less importance. However, a Danish study similarly found an association with greater levels of urbanicity and risk of ASD and an increased risk of ASD in children who moved to a higher level of urbanicity after birth [37]. This could reflect that local resources have some influence on the public attention and health information in the municipalities or that migration is influenced by available treatment options. Our study could not demonstrate this in relation to ADHD diagnosis and treatment although a greater incidence was observed in densely populated areas.

With the possibility of environmental factors influencing the risk of ADHD, children living in different areas would also have different risk exposure of ADHD. However, one problem in evaluating the geographical distribution of ADHD for aetiological purposes lies in difficulties disentangling the geographical distribution of other factors associated with diagnosis. Some factors might promote the recognition of ADHD but not necessarily the occurrence of ADHD.

We cannot, however, rule out the possibility that environmental toxicants have contributed to the geographical variation, but it is unknown if the diagnostic pattern resembles the true occurrence. Also, looking at environmental risk factors, pre- and post-natal exposures to lead and low birth weight/prematurity have been identified as consistent risk indicators, but none are yet known to be definitely causal. ADHD has even been associated to the geospatial factor sunlight [38]. However, Denmark is a rather small country with very little variation in both sunlight and altitude and therefore these factors are probably not important in a Danish context. There is a large amount of literature documenting associations between ADHD and a wide variety of putative environmental risks that can, at present, only be regarded as correlates or potential causes [3]. Therefore, it seems somewhat unlikely that the geographical variation is due to life style or environmental factors alone.

In contrast to studies from the US, we found no statistically significant associations with contextual factors in terms of SES, municipal spending on health care for children or differences in the diagnostic practices. These indicators may, however, be too broad to capture the drivers of diagnostics and treatment. The differences between study results could also be due to different definitions of neighbourhoods. The municipality is the smallest administrative unit in Denmark, but it may be too large to capture the spatial variation of social inequality and too small to capture the regionally organised healthcare system. However, a major strength of this study is the complete follow-up of all citizens; this is a particular strength within health geographics and must be taken into consideration when comparing with studies from countries with incomplete registration where loss to follow-up is likely to provide considerable bias.

Large overdispersion was seen in the non-spatial logistic regression analysis. Overdispersion can be due to clustering (lack of independence) of the incidence proportion of the different indicators of ADHD between municipalities. Significant spatial clustering (by Moran’s I) between municipalities was seen. Overdispersion can also be due to lack of important explanatory variables. This study shows that the variation in ADHD diagnoses and medication use in Denmark is not random as the incidence is highly correlated in the municipalities. One possible cause is confounding if an important spatially correlated covariate is either not measured or unknown [39].

This study used aggregated data at municipal level since we hypothesized that the variation is due to structural factors at this level.

## Conclusions

This exploratory analysis produced maps of the incidence proportions of ADHD by municipality using different indicators. Large variations in the incidence were observed as well as considerable differences in the increase in incidence across municipalities. Significant clustering of both high and low values was identified and mapped using the local Moran’s I. The clustering of low incidence proportions was located in less populated areas with limited diagnostic resources and in contrast clustering of high incidence proportions in densely populated areas with greater diagnostic resources. A large geographical variation of ADHD in the municipalities was observed despite tax-financed and free access to healthcare. Although not statistically significant, results indicate that accessibility to diagnostic resources might explain some of the variation in ADHD incidence. In contrast to US studies the observed variation was not statistically associated to contextual factors in terms of SES, municipal spending on health care for children or differences in diagnostic practices.

With complete follow-up of a whole country’s citizens this study shows that the variation in ADHD diagnosis and medication use is not random and the reasons for the increased incidence of ADHD are probably complex and diverse.

There are likely unknown factors related to diagnostic processes, besides accessibility to diagnostic services that drive the diagnostic occurrence of ADHD.

## Methods

### Study design

We included all children born in Denmark from 1 January 1990 to 31 December 2000. This cohort was extracted from the Danish Medical Birth Registry and consisted of 750,512 children. The Danish Medical Birth Registry comprises data on all live births and stillbirths among women with permanent residence in Denmark [40]. All live born children in Denmark are assigned a unique civil registration number at birth. This makes it possible to link data from the Danish Medical Birth Registry, the Danish Psychiatric Central Register and the Danish National Hospital Register. The registries include information on all inpatient admissions from 1980 and all outpatient contacts to psychiatric hospitals, wards, and clinics in Denmark from 1995 [41, 42]. Inpatient hospital admission corresponds to overnight hospital stays or daily hospital appointments during an extended period for diagnostic evaluation and treatment. Outpatient contacts correspond to less regular appointments. Children with suspected ADHD are generally referred by general practitioners or school psychologists to a paediatric psychiatric ward to undergo diagnostic evaluation and receive treatment. In some cases paediatricians and neurologists take part of the diagnostic evaluation. The Danish Psychiatric Central Register and the Danish National Hospital Register include data on clinical diagnoses, dates of admission and discharge, and reasons for admission, and the International Classification of Diseases, 10th Revision (ICD-10) diagnostic code criteria, which have been used since 1994.

All children were followed from birth until the first diagnosis of ADHD, first use of ADHD medication, death, emigration, the age of 11 years, or 31 December 2011, whichever came first. All children were followed up to the age of 11 years to ensure the same follow-up time of all children as the geographic analysis rely on binomial regression. The last birth cohort in our analyses was born in 2000 as we were able to do a follow-up in 2011. A child was considered to have ADHD if receiving a confirmed diagnosis of ADHD after the age of 5 years or redeeming a prescription for ADHD medication. ADHD can be difficult to diagnose before the age of five therefore the child was only considered a case if registered with a hospital admission related to the diagnosis or redeemed medication after the age of 5 years. Information on ADHD medication was obtained from the Register of Medicinal Product Statistics [43]. A child was considered a case if he/she had redeemed at least two prescriptions for ADHD medication. The ADHD medication included N06BA04 (methylphenidate), N06BA09 (atomoxetine), or N06BA07 (modafinil).

In Denmark, citizens have the right to use privately practicing specialists free of charge if waiting time at public hospital services exceeds 1 month. However, privately practicing psychiatrists are not obligated to report diagnoses to the registries. Thus, we used ADHD medication prescriptions as a proxy for those children who did not have a hospital diagnosis of ADHD. In Denmark, only specialists in paediatric psychiatry are allowed to prescribe ADHD medication to children.

Data were available at individual level. We assigned the cases to the municipality in which they were diagnosed or redeemed prescriptions, whichever came first, while the rest of the study population was assigned to their birth municipality. We performed a sensitivity analysis assigning all children to their birth municipality. The results did not differ from the initial analyses. For the purposes of this analysis we aggregated data and performed the analyses at municipality level. The aggregation was calculated as the incidence proportion for each municipality. The number of cases was divided by the number of children born in the municipality from 1990 to 2000.

The study was approved by the Danish Data Protection Agency.

### Danish health care system

Health care in Denmark is primarily tax-financed and free of charge at the point of service. Danish health care is divided into three political and administrative levels: government level, regional level and municipal level. There are five regions and 98 municipalities in Denmark. The responsibility for running the public health care services is decentralised and divided between the regions and municipalities. The running of secondary care (hospitals) is the responsibility of the five regions. The 98 municipalities are responsible for primary care, public health care, school health service, child dental treatment, prevention and rehabilitation. Costs of most prescriptive medication, including ADHD medication can be reimbursed.

### Spatial autocorrelation

The first phase of the spatial data analysis included mapping the distribution of the different indicators of ADHD incidence proportion; diagnosis, medication use, medication use in children without a registered diagnosis, and overall ADHD (both diagnosis and/or redeeming medication). The next phase included the use of two spatial statistics to determine the spatial clustering: the global and local Moran’s I. The global Moran’s I statistic is a global measure of spatial autocorrelation used to test whether values in a numeric variable are randomly distributed over the geographical area or whether neighbouring values tend to be more similar than non-neighbouring. Moran’s I shows the strength of spatial autocorrelation on a scale ranging from +1 to −1. A value of +1 indicates positive spatial autocorrelation where high values are proximal to other high values. Conversely, a value of −1 represents negative spatial autocorrelation where high values tend to be near low values. A value of zero indicates no spatial autocorrelation, i.e. data are randomly distributed within the studied geographical area. Since global indices of spatial autocorrelation summarize the phenomenon of interest in a single value, they are intended not so much for identifying spatial clusters, as for detecting the presence of a general tendency to clustering within the study area. The local Moran’s I statistic reveals whether and where any local clustering occurs. The local Moran’s I identifies individual clusters, or small regions of clusters that may not be evident within the global pattern [44].

An analytic tool in STATA developed by Maurizio Pisati was used to calculate both the global and the local Moran’s I statistic and associated Z-score. This tool tests whether the homogeneity (or heterogeneity) in values between a municipality and its neighbouring municipalities is higher than would be expected by chance. A municipality with a high Moran’s I statistic indicates that its incidence proportion values are close in magnitude to those of the neighbouring municipality. We used the tool to draw cluster maps visualising what is also called “hot spots” (correlation of municipalities with high incidence proportion values) and “cold spots” (correlation of municipalities with low incidence proportion values) [45].

The analysis of spatial autocorrelation requires a measurement of the degree of spatial proximity among the spatial objects of interest. Typically, the degree of spatial proximity among a given set of spatial objects is represented by a matrix called spatial weights matrix (W) [44]. We used the common variant of W the row-standardized spatial weights matrix W_std_. The distance band was chosen to be 200 km as the median distance between the centroids in the municipalities was 141 km and the 3rd quartile distance was 200 km. We performed a sensitivity analysis with distance bands of 150 and 300 km; this did not significantly change the clusters. The only changes were the appearance of one municipality and the disappearance of another in clusters with low incidence proportions.

A two sided p value of <0.05 was considered statistically significant.

### Municipality-level contextual factors

To examine access to diagnostic services, information on number and municipality of privately practicing paediatric psychiatrists was extracted from a national register on provision of privately practicing physicians in 2009. The variables concerning economic characteristics in the municipalities were derived from Statistics Denmark for 2010 providing information aggregated at municipality level. The measures of SES in the municipalities and the municipal spending on health care for children reflect the local resources spent in the municipalities. The municipal spending on primary health care for children is a measure of the services provided by health visitors and school nurses. These services are available for all Danish children and can be used as a measure of the capacity of paediatric health promotion and disease prevention at municipality level. The average annual household income was used as a proxy for municipality level SES and calculated as the sum of the main source of income. The average municipal spending on primary health care for children was calculated as the net operating costs per child aged 0–16 years per year including the average costs of primary care including school nurse and health visitor services. Differences in diagnostic culture were examined by studying variation in the incidence proportion of conduct disorders. A number of criteria for ADHD are also considered a sign of conduct disorder and there might be a difference in attitude to diagnosing. If the peadiatric psychiatrist diagnose a child with ADHD he accepts at the same time that the cause is primarily genetically causing biological dysfunctions in the brain and therefore the treatment is primarily medical. If instead the psychiatrist believes that the childs problems are mainly environmental he would think that the child is suffering from behavioral problems such as conduct disorder. We hypothesised that the incidence proportion of conduct disorders may be higher in municipalities with a lower ADHD incidence proportion reflecting the difference in the professional’s beliefs and decision-making. The distribution of the incidence proportions of the ICD-10 hospital diagnoses F.91 conduct disorders and F.92 mixed disorders of conduct and emotions served as a proxy for diagnostic culture.

### Non-spatial regression of incidence proportions

A non-spatial logistic regression analysis was initially performed to examine the associations between incidence proportions of children with diagnosis, medication use without a registered hospital diagnosis and overall ADHD and resources. The number of children with a diagnosis, medication use without a registered diagnosis and overall ADHD (both diagnosis and/or medication) were analysed separately. The number of children with diagnosis, medication use and ADHD, respectively, were outcomes. Absence of a hospital or paediatric psychiatrist in the municipality (yes, no), household income, average municipal spending on primary health care for children and incidence proportion of conduct disorders (100 × (count F91 + count F92/N)) were included as fixed effects (explanatory variables). Pearson correlation analysis was performed to check multicolinearity of the explanatory variables. The assumption about linearity between each outcome and the explanatory variables of average yearly total household income, average municipal spending on primary health care for children and proportion of children with a conduct disorder diagnosis was evaluated by categorising the explanatory variables and examining if the estimates of the categorised variable indicated linearity. The explanatory variables were categorised in three and four groups, respectively. Furthermore, linearity was evaluated by including the explanatory variable as a continuous variable as linear and quadratic terms in the analysis. If the quadratic term was significant it indicated that the assumption about linearity was not confirmed. The assumption about linearity for the three outcomes was not confirmed and the three explanatory variables were each categorised into three equally sized groups (low, medium, high).

The logistic regression model used was:

$${\text{logit}}\left( {\pi_{i} } \right) = \mu + I_{i} + R_{j} + B_{k} + P_{l}$$

where $\pi_{i}$ is the proportion of children with diagnosis, medication use without a registered diagnosis or overall ADHD in municipality $i$, *i* = 1,…, *n*, and *n* = 98.

$\mu$ is the intercept.

$I_{i}$ is the fixed effect of municipal average of yearly total household income, *i* = low, medium, high.

$R_{j}$ is the fixed effect of the municipal spending, *j* = low, medium, high.

$B_{k}$ is the fixed effect of municipal incidence proportion of conduct disorder *k* = low, medium, high.

$P_{l}$ is the fixed effect of municipal absence of hospital or paediatric psychiatrist, *l* = yes, no.

Significance of explanatory variables was evaluated using a likelihood ratio test. Model fit was evaluated using Pearson dispersion parameter. Adjustment for over-dispersion was performed based on Pearson goodness-of-fit statistics. Overdispersion is the presence of greater variability in a data set than would be expected based on the statistical model.

The logistic regression analyses were performed in Statistical Analysis Software package (SAS, version 9.3).

### Bayesian conditional autoregressive analysis of the incidence proportions

Areal data typically exhibit spatial autocorrelation with observations from areal units close together tending to have similar values. A proportion of this spatial autocorrelation may be modelled by including known covariate risk factors in a regression model. It is, however, common for spatial structure to remain in the residuals after accounting for these covariate effects. This residual spatial autocorrelation can be induced by a number of factors, and violates the assumption of independence common in many regression models. Bayesian modelling produces parameter estimates for each individual analysis unit by borrowing information from all other analysis units [39]. A Bayesian conditional autoregressive (CAR) analysis was performed to evaluate the significance of the explanatory variables when considering the spatial autocorrelation between neighbouring municipalities and non-spatial variation for each municipality not accounted for by the explanatory variables [39]. A Bayesian CAR model with a binomial distribution of number of children with diagnosis, medication use without a registered diagnosis and overall ADHD were analysed separately. Presence of a hospital or paediatric psychiatrist in the municipality (yes, no), household income, average municipal spending on primary health care for children and incidence proportion of conduct disorders were included as fixed effects (explanatory variables). The CAR model suggested by Leroux was used [46]. The CAR analysis models overdispersion and spatial autocorrelation in data present after adjusting for the explanatory variables. Spatial correlation between neighbouring municipalities is modelled by a 98 × 98 neighbourhood (adjacency) matrix, whose *jk*th element is 1 if the municipalities *j* and *k* are sharing a common border, otherwise 0.

The Bayesian CAR model used was:

$${\text{logit}}\left( {\pi_{i} } \right) = \mu + I_{i} + R_{j} + B_{k} + P_{l} + \varphi_{i}$$

where $\pi_{i}$, $\mu$, $I_{i}$, $R_{j}$, $B_{k} , P_{l}$ are defined as above.

$\varphi_{i}$ is the random effect of municipality $i$, *i* = 1,…, *n*, and *n* = 98.

A weakly informative independent Gaussian prior (N(0, 10^2^)) was specified for parameters of the explanatory variables.

The spatial autocorrelation was modelled using Leroux model as the CAR prior given by $\varphi_{i} \sim N\left( {\frac{{\rho \mathop \sum \nolimits_{j = 1}^{n} w_{ij} \varphi_{j} }}{{\rho \mathop \sum \nolimits_{j = 1}^{n} w_{ij} + 1 - \rho }} , \frac{{\tau^{2} }}{{\rho \mathop \sum \nolimits_{j = 1}^{n} w_{ij} + 1 - \rho }}} \right)$ when conditioning on all other municipalities, where $\rho$ is the spatial correlation between neighbouring municipalities.

$\mathop \sum \nolimits \varphi_{j}$ is the sum of the random effects of neighbouring municipalities.

$w_{ij}$ is 1 if municipalities *i* and *j* are neighbours (i.e. share a common border) and 0 otherwise.

$\tau^{2}$ is the random variation not accounted for by the explanatory variables.

The variance parameter $\tau^{2}$ was assigned a uniform prior U(0,1000) and the spatial autocorrelation parameter $\rho$ was assigned a uniform prior U(0,1).

Inference (parameter estimation) for this model was based on Markov chain Monte-Carlo (MCMC) simulation, using a combination of Gibbs sampling and Metropolis steps. A single chain was applied. Each model was estimated with 100,000 iterations for burn-in and 1,000,000 iterations with thinning = 100.

Significance was assessed as non-zero parameter estimates using 95 % credible intervals.

Residuals of the final model for each outcome were mapped to evaluate how well the models performed. Convergence was evaluated by plotting the samples of selected parameters. The spatial regression analyses were performed in R using the CARBayes package (R version 3.1.2).

## Abbreviations

ADHD: attention-deficit/hyperactivity disorder
DSM-IV: diagnostic and statistical manual of mental disorders 4th edition
ICD-10: International classification of diseases 10th revision
SES: socioeconomic status
